# A Unique Bivalent Binding and Inhibition Mechanism by the Yatapoxvirus Interleukin 18 Binding Protein

**DOI:** 10.1371/journal.ppat.1002876

**Published:** 2012-08-23

**Authors:** Brian Krumm, Xiangzhi Meng, Zhixin Wang, Yan Xiang, Junpeng Deng

**Affiliations:** 1 Department of Biochemistry and Molecular Biology, Oklahoma State University, Stillwater, Oklahoma, United States of America; 2 Department of Microbiology and Immunology, University of Texas Health Science Center at San Antonio, San Antonio, Texas, United States of America; Saint Louis University, United States of America

## Abstract

Interleukin 18 (IL18) is a cytokine that plays an important role in inflammation as well as host defense against microbes. Mammals encode a soluble inhibitor of IL18 termed IL18 binding protein (IL18BP) that modulates IL18 activity through a negative feedback mechanism. Many poxviruses encode homologous IL18BPs, which contribute to virulence. Previous structural and functional studies on IL18 and IL18BPs revealed an essential binding hot spot involving a lysine on IL18 and two aromatic residues on IL18BPs. The aromatic residues are conserved among the very diverse mammalian and poxviruses IL18BPs with the notable exception of yatapoxvirus IL18BPs, which lack a critical phenylalanine residue. To understand the mechanism by which yatapoxvirus IL18BPs neutralize IL18, we solved the crystal structure of the Yaba-Like Disease Virus (YLDV) IL18BP and IL18 complex at 1.75 Å resolution. YLDV-IL18BP forms a disulfide bonded homo-dimer engaging IL18 in a 2∶2 stoichiometry, in contrast to the 1∶1 complex of ectromelia virus (ECTV) IL18BP and IL18. Disruption of the dimer interface resulted in a functional monomer, however with a 3-fold decrease in binding affinity. The overall architecture of the YLDV-IL18BP:IL18 complex is similar to that observed in the ECTV-IL18BP:IL18 complex, despite lacking the critical lysine-phenylalanine interaction. Through structural and mutagenesis studies, contact residues that are unique to the YLDV-IL18BP:IL18 binding interface were identified, including Q67, P116 of YLDV-IL18BP and Y1, S105 and D110 of IL18. Overall, our studies show that YLDV-IL18BP is unique among the diverse family of mammalian and poxvirus IL-18BPs in that it uses a bivalent binding mode and a unique set of interacting residues for binding IL18. However, despite this extensive divergence, YLDV-IL18BP binds to the same surface of IL18 used by other IL18BPs, suggesting that all IL18BPs use a conserved inhibitory mechanism by blocking a putative receptor-binding site on IL18.

## Author Summary

Interleukin 18 (IL18) is an important cytokine in inflammation and immunity. Mammals and poxviruses encode homologous inhibitory proteins of IL18, named IL18BPs, which regulate IL18 activity and, in the case of the viral proteins, contribute to virulence. Previous structural and functional studies revealed residues at IL18:IL18BP interface that are critical for the high-affinity binding, including a phenylalanine on IL18BPs, which is conserved among nearly all IL18BPs with the notable exception of yatapoxvirus IL18BPs. To understand the mechanism by which yatapoxvirus IL18BPs neutralize IL18, we solved the high-resolution crystal structure of the Yaba-Like Disease Virus (YLDV) IL18BP:IL18 complex. The structure revealed a 2∶2 bivalent binding complex, which has not been observed in any other IL18BPs. Through mutagenesis and functional studies, we found a set of interacting residues that are unique for the association of YLDV-IL18BP and IL18, likely compensating for the lack of the interactions involving the conserved phenylalanine. Despite this extensive divergence, however, YLDV-IL18BP binds to the same surface of IL18 used by other IL18BPs. Our study suggests that all IL18BPs use a conserved inhibitory mechanism by blocking a putative receptor-binding site on IL18 but the interface on IL18 is malleable by a broad and diverse family of mammalian and poxvirus IL18BPs.

## Introduction

Poxviruses are a family of large, complex DNA viruses, infecting a variety of organisms including insects, reptiles, birds and mammals [1]. The poxvirus family is further subdivided into genera based on shared characteristics such as host range, morphology, antigenicity, and sequence similarity [2]. Four genera of poxviruses are known to be pathogenic to humans, including molluscipoxvirus, orthopoxvirus, parapoxvirus, and yatapoxvirus. As an immune evasion strategy, poxviruses encode an assortment of decoy receptors for chemokines and cytokines [3]. One such strategy for evasion of the host immune response is through modulation of the interleukin 18 (IL18) signaling pathway. IL18 is a pro-inflammatory cytokine belonging to the interleukin 1 superfamily and plays an important role in both innate and acquired immune responses by inducing interferon-γ (IFN-γ) production from T lymphocytes and macrophages while also enhancing the cytotoxicity of natural killer cells [4]. IL18 activity is modulated *in vivo* by a negative feedback mechanism involving a naturally occurring IL18 inhibitor, the IL18 binding protein (IL18BP) [5]. Homologues of IL18BPs are also encoded by many poxviruses including molluscum contagiosum virus and orthopoxviruses [6], [7] such as variola virus, the causative agent of smallpox.

Yaba-Like Disease Virus (YLDV) along with Yaba Monkey Tumor Virus (YMTV) are members of the yatapoxvirus genus of poxviruses. These viruses produce a very distinct disease in primates that is characterized by epidermal histiocytomas and vesicular lesions of the head and limbs [8]–[10]. Although their exact host reservoir is not well established, it is presumed that the immunomodulatory proteins expressed by these viruses can at least partially cope with the primate/human immune system. Analysis of YLDV and YMTV genome revealed yatapoxviruses encode a predicted IL18BP family member, designated as 14L [11], [12]. YLDV and YMTV 14L proteins share approximately 54% sequence identity between each other but less than 14% with IL18BPs of the orthopoxviruses such as the variola virus and the mousepox ectromelia virus (ECTV). Despite this low sequence similarity, YMTV 14L was previously shown to be a functional IL18 inhibitory protein with comparable affinity as orthopoxvirus IL18BPs [13].

**Figure 1:**
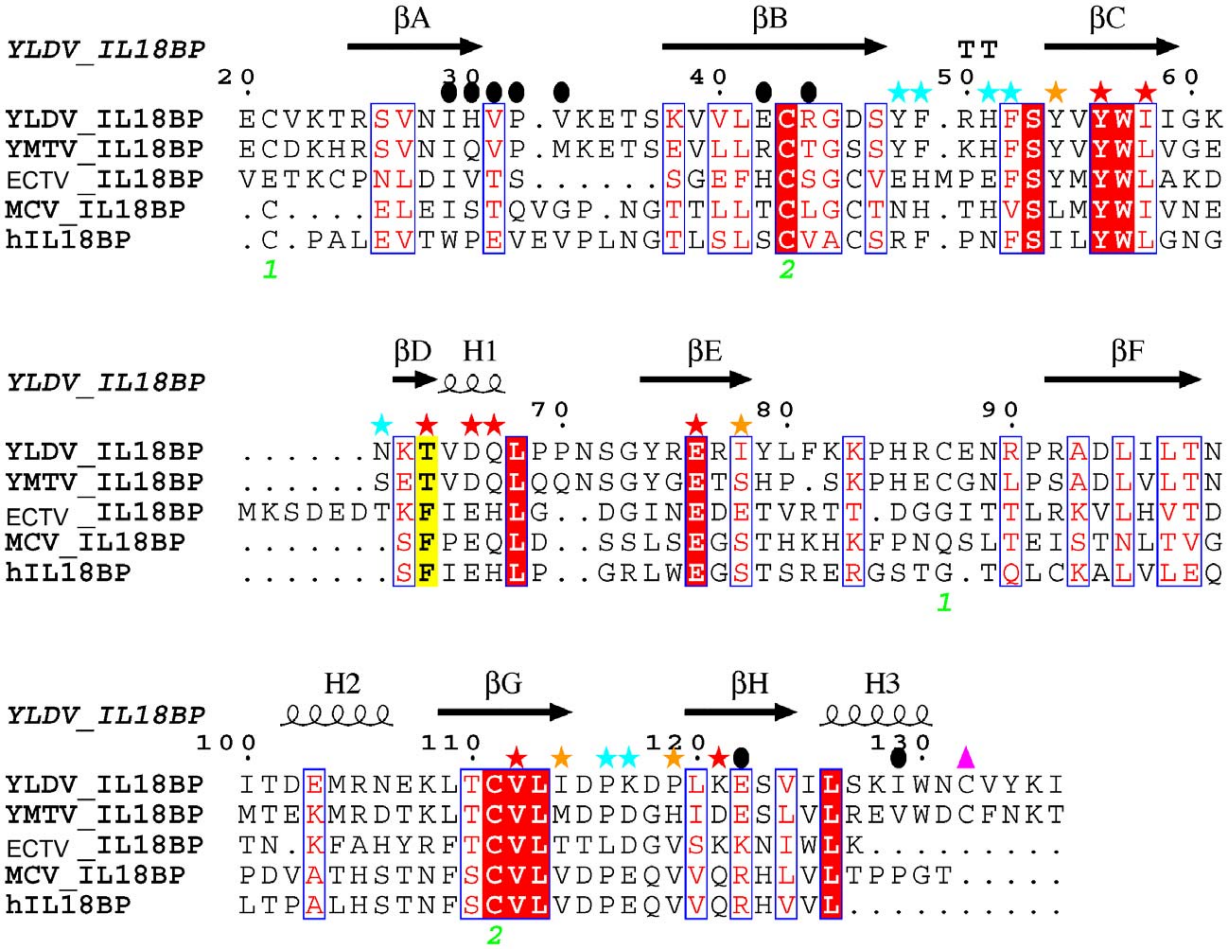
Structure based sequence alignment of IL18 binding proteins. Structure based sequence alignment of various IL18BPs was created using the crystal structure of YLDV-IL18BP as the template. Lettering and numbering above alignment correspond to YLDV-IL18BP topology and numbering scheme. Colored stars above residues indicate the three binding sites on IL18 with which they interact, red: site A, orange: site B, cyan: site C. Solid black circles above residues indicate resides involved in homo-dimerization. The two intra-chain disulfide bonds are indicated with the green letters. The cysteine residue forming the unique inter-chain disulfide bond is indicated with a pink triangle. The sequence alignment was performed with the FATCAT server [44] and ClustalX [45], and the figure was created with ESPript [46].

The high-resolution crystal structure of the ECTV-IL18BP in complex with human IL18 revealed the structural basis by which orthopoxvirus IL18BPs antagonize IL18 signaling through direct competition with IL18 cognate receptor for binding [14]. The crystal structure along with mutagenesis studies identified a set of conserved residues from IL18 and IL18BPs as key to complex formation. In particular, a phenylalanine (F67 in ECTV-IL18BP) residue that is highly conserved in IL18BPs was found indispensible for IL18 binding [14]–[17]. Mutations of this site in all IL18BPs examined to date significantly decreased or even completely abolished the binding to IL18. In addition, a residue on IL18, K53, was shown as a ‘hot spot’ for binding IL18BPs, since mutations at this site drastically decreased binding affinity [18]. A strong π-cation interaction between IL18 K53 and the conserved phenylalanine residue (F67) of ECTV-IL18BP was revealed in the structure of ECTV-IL18BP:IL18 complex, explaining its important role in binding. Surprisingly, phylogenetic analysis and sequence alignment revealed the presence of a threonine (T64) in yatapoxvirus IL18BPs at the position equivalent to the conserved phenylalanine (Figure 1) [19]. Furthermore, mutation of K53 on IL18 only modestly affected the binding with YMTV 14L [13]. To understand how yatapoxvirus IL18BPs bind IL18, we determined the high-resolution crystal structure of YLDV-IL18BP:IL18 complex. This structure along with the functional analysis through mutagenesis and Surface Plasmon Resonance (SPR) provide new insights into the mechanism by which IL18BPs inhibit IL18. The result provided here could be helpful for developing inhibitors for IL18 or IL18BP.

## Results

### Architecture of the YLDV-IL18BP:IL18 Complex: Bivalent Binding

Recombinant human IL18 and mature YLDV-IL18BP (residue 20–136) proteins were individually purified from *E. coli*, and were subsequently used to reconstitute a complex of IL18:YLDV-IL18BP. Initially, wild-type (WT) IL18 and YLDV-IL18BP were used, but no quality crystals were obtained. In efforts to improve crystallization, we mutated some non-essential cysteines and additional surface residues in IL18. Substitution of four cysteines with serines in IL18 was previously shown to increase the stability of IL18 without affecting IL18 activities [20]. We found that the use of an IL18 mutant [IL18 (8S), see Materials and Methods], containing substitutions of the four-cysteines with serines and substitutions of four surface residues opposite to the IL18BP binding interface with alanines, greatly improved crystal quality and reproducibility. The crystal structure of IL18 (8S) in complex with YLDV-IL18BP was determined to 2.7 Å. Furthermore, a crystal from the complex of IL18 (8S) and a three-cysteine mutant of YLDV-IL18BP (ΔC21, C87S, C132S) diffracted to 1.75 Å (see Materials and Methods, Table 1). The two structures are essentially identical to each other with a root mean square deviation (r.m.s.d) of less than 0.4 Å, and the three mutated cysteines in YLDV-IL18BP are distant from the IL18 binding interface. Therefore, for discussion of protein:protein interactions between YLDV-IL18BP and IL18, we will mainly focus on the higher resolution structure of the mutant YLDV-IL18BP.

**Table 1:** X-Ray crystallographic data and refinement statistics.

	YLDV-IL18BP(WT):IL18 (8S) (WT)	YLDV-IL18BP(3CM):IL18 (8S)* (Triple Mutant)
Beamline	19-ID, APS	X29, BNL
Wavelength, Å	0.97911	1.0750
Space Group	P2_1_	P2_1_
Cell Parameters: a, b, c (Å); β (°) (°) (°)	76.4,41.8,104.4; 98.3	75.4,41.2,100.8; 97.9
Resolution, Å	50 - 2.7	50 - 1.75
Total Reflections	349,408	504,980
Unique Reflections	18,239 (1,814)	61,263 (2,873)
Redundancy	3.6 (3.4)	3.7 (3.6)
Completeness, %	100 (100)	98.1 (93.0)
I/σ	10.8 (1.8)	34.6 (1.8)
R_sym_, %	12.2 (67.8)	5.3 (69.6)
**Refinement Statistics**		
Resolution range used, (Å)	37.3-2.7	33.2-1.75
No. reflections used	34,563	61,221
*R*_work_/*R*_free_	21.9/27.0	19.0/23.1
Rmsd bond lengths (Å)	0.006	0.007
Rmsd bond angles (°)	0.904	1.035
Number of atoms		
Protein	4,227	4,108
Water	25	274
Ramachandran Values		
Preferred regions (%)	93.6	97.3
Allowed regions (%)	6.4	2.7

* WT, wild type; 3CM, triple-cysteine mutant.

Values in parentheses are for the highest resolution shell, 2.80 to 2.70 Å (WT), 1.78 to 1.75 Å (3CM).

R_sym_ = Σ |*I*_obs_−*I*_avg_|/Σ *I*_avg_; *R*_work_ = Σ‖ *F*_obs_ |−|*F*_calc_‖/Σ *F*_obs_.

*R*_free_ was calculated using 10% and 5% of data for the WT and 3CM complexes, respectively.

**Figure 2:**
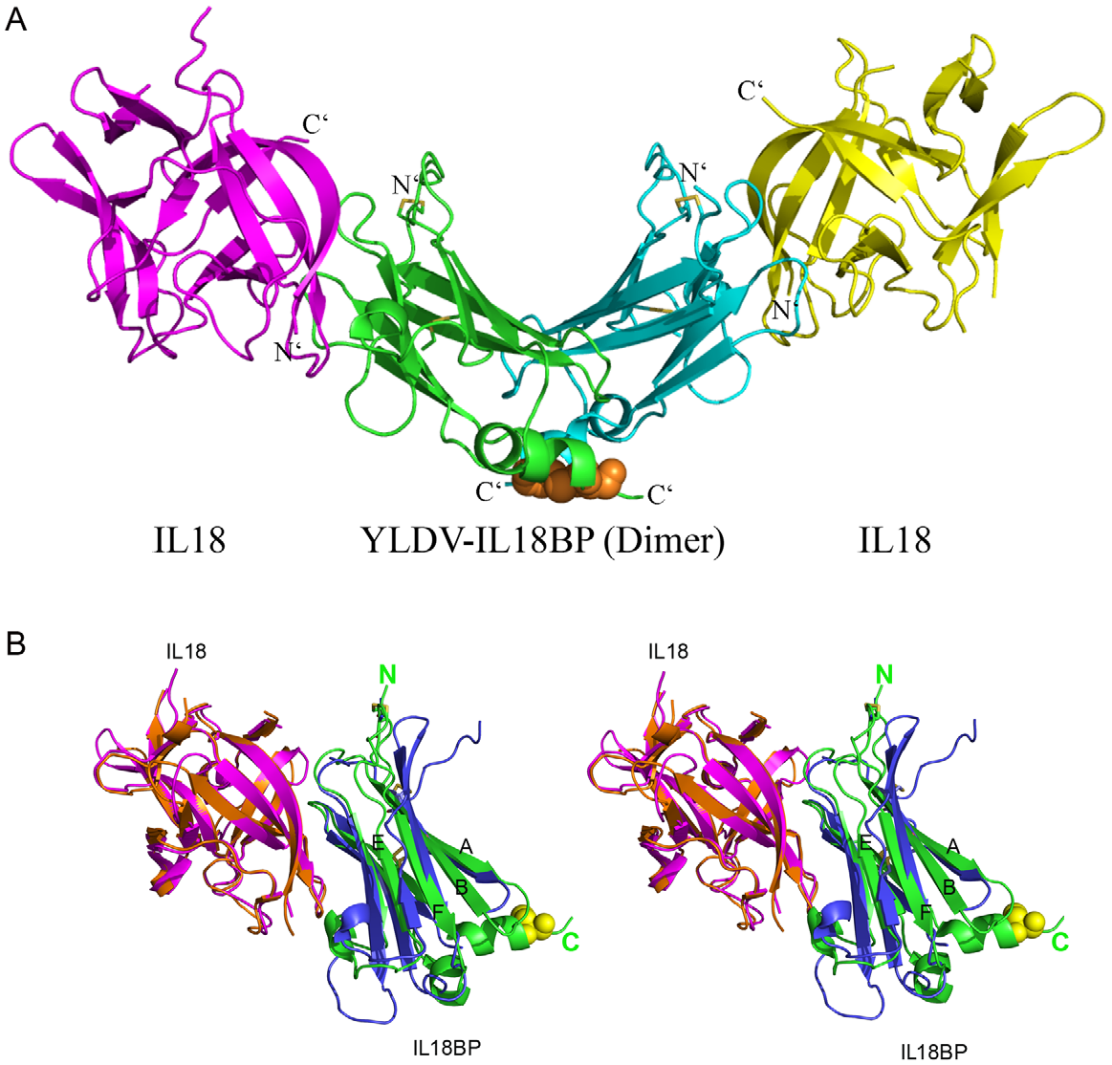
Overall structure of YLDV-IL18BP:IL18 complex. A). YLDV-IL18BP displays a homo-dimer (green and cyan), each adopting a β-sandwiched Ig-fold structure. Each protomer binds one molecular of IL18 (yellow and magenta), forming a 2∶2 complex. The intra-chain disulfide bonds (SS) in YLDV-IL18BPs are shown as yellow sticks. The inter-chain SS bond at the C-terminus of YLDV-IL18BPs is shown as orange spheres. B). Stereoview of superimposition of the monomer complexes of YLDV-IL18BP (green):IL18 (magenta) and ECTV-IL18BP (blue):IL18 (orange). The N- and C-terminus of YLDV-IL18BP are indicated. The intra-chain SS bonds are shown as in A. Residue C132 is shown as yellow spheres. The β-strands A, B, E and F in YLDV-IL18BP are labeled.

The structure of the complex shows that the YLDV-IL18BP forms a homo-dimer in a back-to-back fashion, with each protomer binding to a molecule of IL18, forming a hetero-tetramer complex with a stoichiometry of 2∶2 (Figure 2A). The complex displays an elongated v-shaped architecture with the IL18BP homo-dimer at the center and IL18 at the ends.

As seen in the ECTV-IL18BP:IL18 complex [14], IL18 adopts the same β-trefoil fold, which is comprised of 12 β-strands (β1–β12) with one short α-helix and one 3_10_-helix. The IL18 molecules in the two structures show very little conformational changes, with an r.m.s.d. of only 0.5 Å from 134 aligned IL18 Cα backbones (Figure 2B). The two IL18 molecules in the current complex structure are also nearly identical, having only a 0.1 Å r.m.s.d from 150 aligned cα backbone residues.

**Figure 3:**
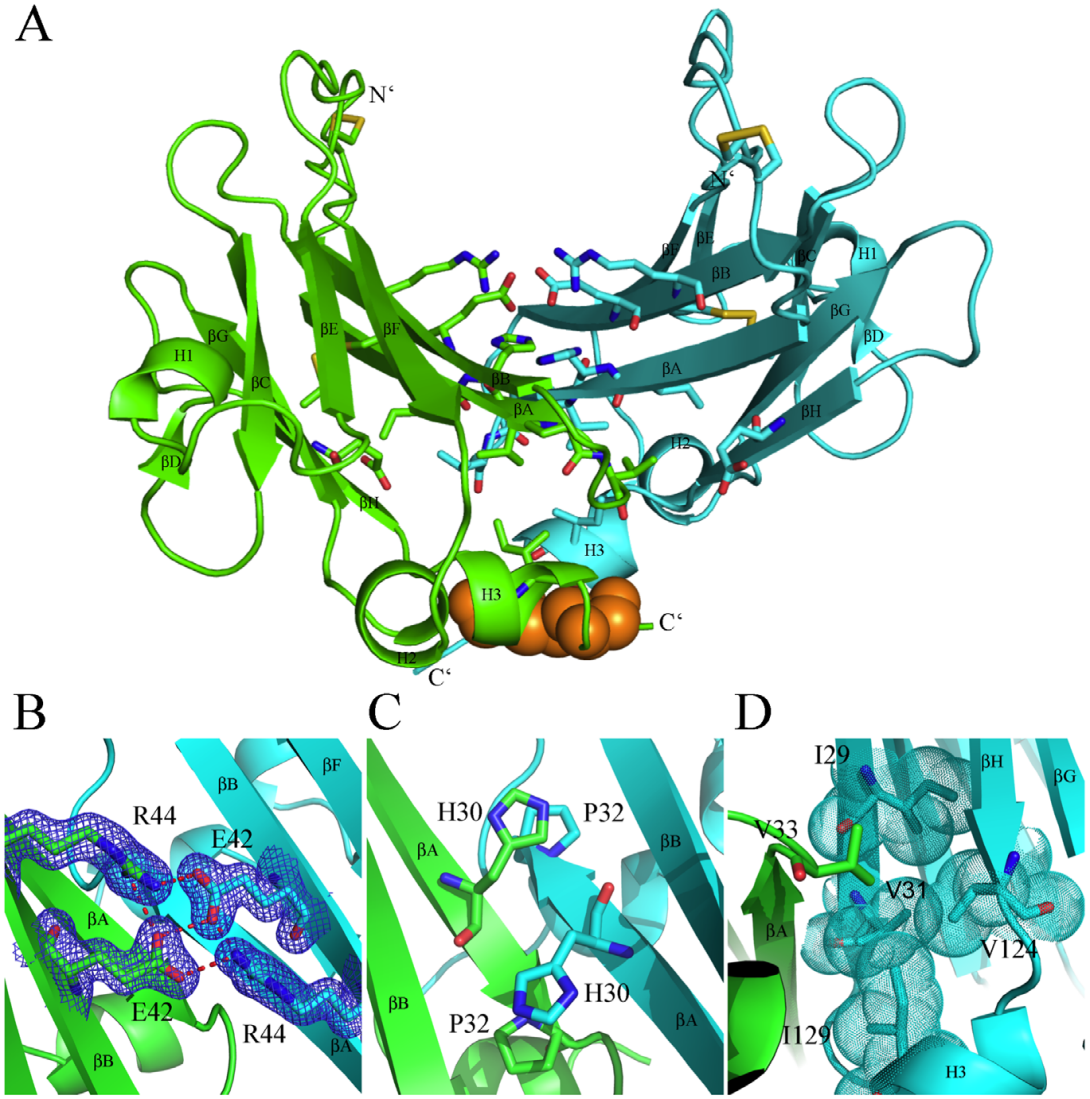
Key interactions of the YLDV-IL18BP homo-dimer. A). YLDV-IL18BP dimerizes back-to-back, abutting on one edge of the Ig-fold through extensive hydrophobic interactions. The secondary structures are labeled. The inter-chain C132-C132 SS bond is shown as orange spheres. B). The E42 from one protomer appears to be protonated, forming a hydrogen bond with E42 from the other protomer. E42 also forms both intra-chain and inter-chain hydrogen bonds with R44. E42 and R44 residues are shown as sticks. The *2mFo-DFc* electron density map is shown in blue. C). Hydrophobic stacking of residues H30 and P32 of one YLDV-IL18BP protomer with their counterparts of the other protomer. D) Residue V33 of one protomer is inserted into the hydrophobic platform comprised of I29, V31, V124, and I129 of the second protomer.

Each protomer of YLDV-IL18BP adopts a canonical h-type immunoglobulin (Ig) fold [21] comprised of mainly β-sheets as observed previously for ECTV-IL18BP (Figure 3A). However, there is an r.m.s.d. of 2.9 Å over the 106 aligned cα backbone residues between the two viral IL18BPs. ECTV-IL18BP has an extended β-sheet architecture with predominantly shorter loops connecting the β-sheets and β-strands, while YLDV-IL18BP has comparatively shorter β-sheets with extended connecting loops. The two IL18BP structures differ mostly at one sheet of the β-sandwich fold (βA, βB, βE and βF) that is not involved in binding IL18 (Figure 2B). There is a major rigid-body movement on this β-sheet, especially at strands βA, βE and βF where twists are estimated at about 30 to 45 degrees. Compared to ECTV-IL18BP, there are two additional α-helices located between β-strands F and G (H2), and at the C-terminus (H3). YLDV-IL18BP contains five cysteine residues, forming two intra-molecular disulfide bonds (SS) (C21–C87, C43–C111) and one inter-molecular SS bond (C132–C132), in contrast to only two intra-molecular SS bonds in ECTV-IL18BP. C43–C111 is the conserved SS bond among many Ig-fold proteins, which connects the two β-sheets and plays a key role in maintaining overall integrity of the Ig-fold structure. C21–C87 SS bond connects the very N-terminus prior to βA with the loop between βE–βF. In contrast, the very N-terminus prior to βA of ECTV-IL18BP is SS bonded to the neighboring βB. In the triple-cysteine mutant YLDV-IL18BP:IL18 structure, the βE–βF loop is not visible in the electron density map, suggesting that C21–C87 SS bond stabilizes the local structure, particularly the βE–βF loop (Figures 2, 3A). However, the C21–C87 SS bond is not critical for the overall structure of YLDV-IL18BP or its binding with IL18 (shown later).

### Back-to-Back Dimer of YLDV-IL18BP

**Figure 4:**
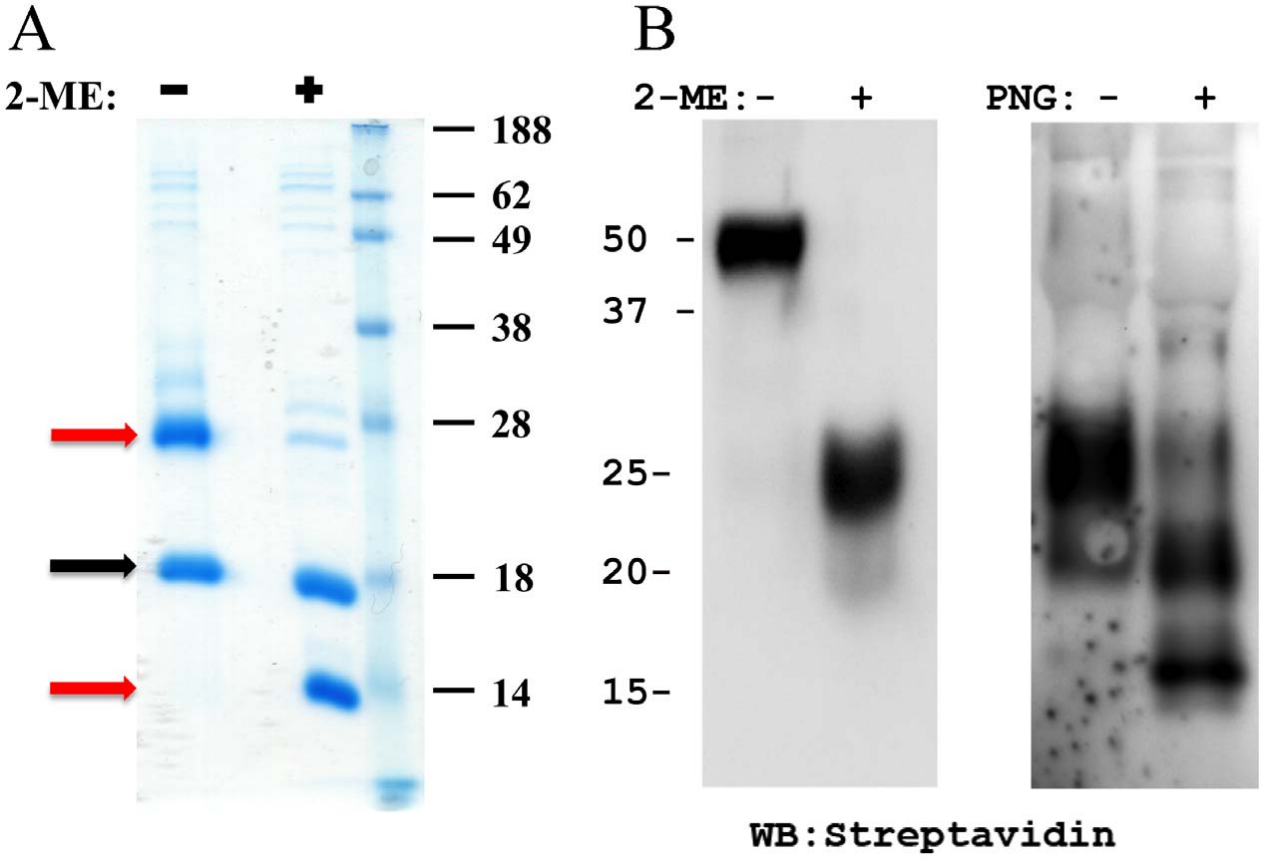
YLDV-IL18BP forms a SS linked dimer in solution. SDS-PAGE analysis of purified YLDV-IL18BP proteins in the presence (+) or absence (−) of 2-mercaptoethanol (2-ME). A). Purified YLDV-IL18BP:IL18 protein complex that was expressed in *E. coli*. IL18 and YLDV-IL18BP are indicated by black and red arrows, respectively. B). Purified YLDV-IL18BP that was expressed and secreted from mammalian cells. The YLDV-IL18BP was engineered with a C-terminal site-specific biotinylation site, which allows its detection in Western blot with streptavidin. Two N-glycosylation sites were predicted for YLDV-IL18BP. Panel B also shows the SDS-PAGE of YLDV-IL18BP that was untreated (−) or treated (+) with endoglycosidase PNGase F.

**Figure 5:**
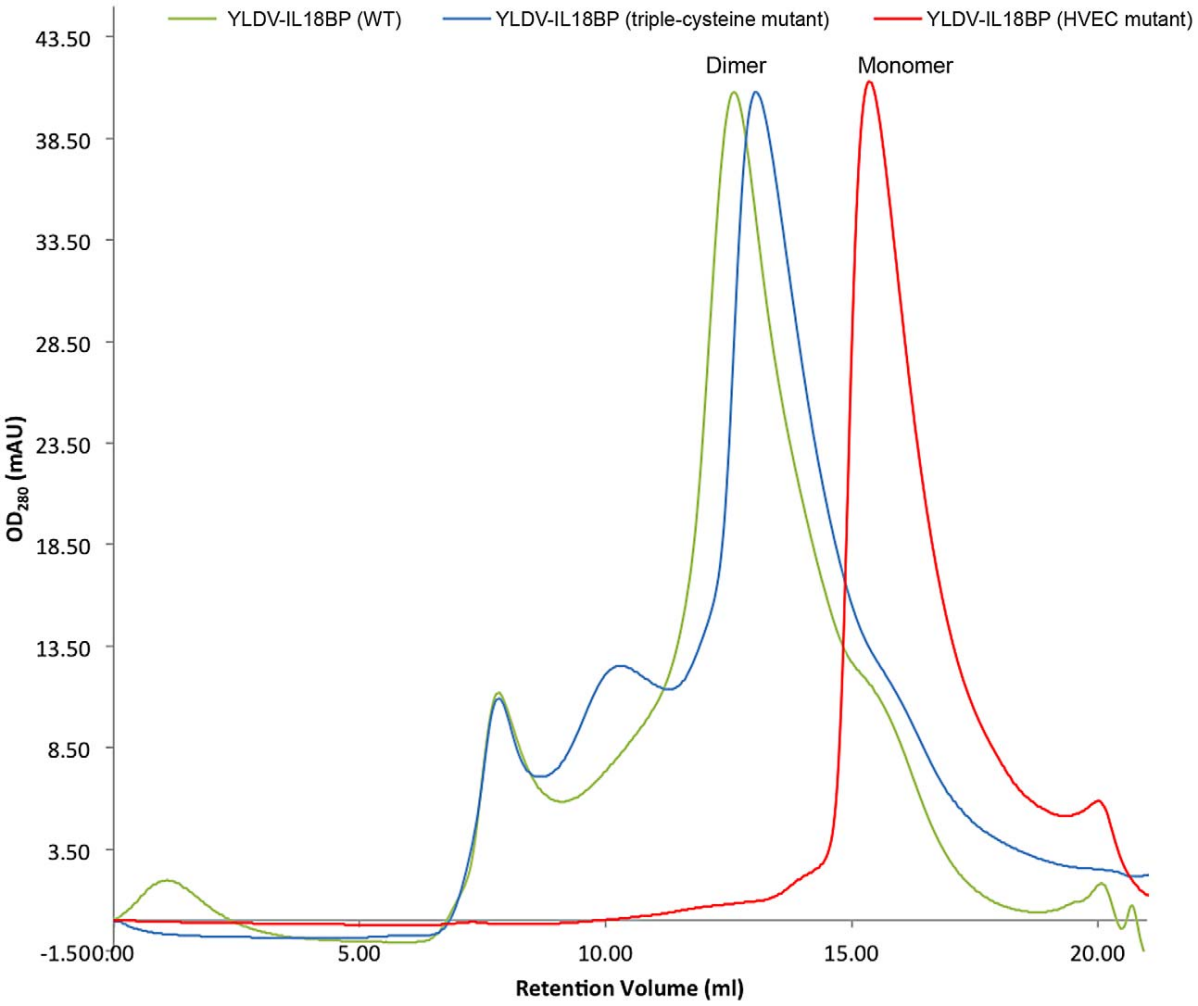
Size exclusion chromatography analysis of YLDV-IL18BP. Various SUMO fusion proteins containing WT, triple-cysteine (ΔC21, C87S, C132S) mutant and the HVEC (H30A/V33R/E42R/C132S) mutant YLDV-IL18BPs were purified and analyzed on a Superdex S200 sizing column. Both WT (Green) and triple-cysteine mutant (blue) display as a dimer, with a calculated MW about 65 kDa estimated from the retention volumes. The HVEC mutant (red) displays predominantly as a monomer, with an estimated MW about 31 kDa. The minor peaks were identified from SDS-PAGE analysis as impurities unrelated to YLDV-IL18BP.

YLDV-IL18BP dimerizes back-to-back, abutting on one edge of the β sandwich, exposing the opposite edge for binding IL18 (Figures 2,3). The homo-dimer interface involves mainly βA, βB, βH and the C-terminal helix H3. It involves extensive hydrophobic interactions in the center (I29, H30, V31, P32 and V33 from βA, the aliphatic side chain of E122, V124 from βH and I129 from H3) flanked by hydrogen bonding and charge-charge interactions, burying approximately 1,700 Å^2^ and 1,465 Å^2^ solvent accessible surface area (ASA) for the wild-type complex and the triple-cysteine mutant complex, respectively (Figure 3). The lack of the C132-C132 SS bond in the triple-cysteine mutant complex caused the disorder of four residues at the C-terminus beyond S132, resulting in a slightly smaller ASA. At the dimer interface, the imidazole ring of H30 from one protomer stacks on the P32 from the other (Figure 3C), while V33 is forming favorable van der Waals interactions with a hydrophobic platform comprised of I29, V31, V124 and I129 from the other protomer (Figure 3D). E42 from one protomer appears to be protonated, forming favorable hydrogen bonds with E42 from the other protomer. E42 also forms both intra-chain and inter-chain hydrogen bonds with R44 (Figure 3B). The inter-chain C132-C132 SS bond covalently links the two promoters. Indeed, a non-reducing SDS-PAGE showed that YLDV-IL18BP proteins expressed in *E. coli* or in mammalian cells formed disulfide-bonded dimers (Figure 4). However, the triple-cysteine mutant of YLDV-IL18BP displays nearly identical structure as the WT protein, and it exists as a dimer in solution judging by size exclusion chromatography (Figure 5) and dynamic light scattering analysis (data not shown). Therefore, the dimerization is not solely dependent on the C132-C132 inter-chain SS bond.

We performed additional mutagenesis studies to verify the importance of the residues at the homo-dimerization interface. E42R/C132S (EC) mutant remained a dimer in solution as the WT (data not shown), while mutants bearing H30A/V33R/C132S (HVC) substitutions or H30A/V33R/E42R/C132S substitutions (HVEC) appeared as monomers in solution based on size exclusion chromatography (Figure 5) and dynamic light scattering analysis (data not shown). Therefore, hydrophobic interactions as well as the disulfide bonding together contribute to the dimerization. When measuring the binding affinity with IL18 by Surface Plasmon Resonance (SPR), we found that the monomeric YLDV-IL18BP (HVEC) had a 3-fold decrease (student t-test P-value<0.05) in binding affinity than the dimeric WT YLDV-IL18BP (Table 2).

**Table 2:** Kinetics and affinity constants of the binding of YLDV-IL18BP mutants with immobilized IL18.

YLDV-IL18BP mutants^a^	K_On_,10^5^/Ms	K_off_,10^−4^/s	K_D_, nM
WT (dimer)	1.8±0.1	0.9±0.2	0.4±0.1
HVEC (monomer)	1.8±0.1	2.4±0.4	1.3±0.1
F52A(monomer)	1.3±0.3	2.6±0.6	2.0±0.1
Y54A(monomer)	1.4±0.6	1.6±0.8	1.1±0.1
Y56A(monomer)	NB^b^		
T64A(monomer)	1.1±0.1	3.6±0.5	3.2±0.1
T64F(monomer)	1.3±0.1	2.0±0.1	1.7±0.1
Q67A(monomer)	1.4±0.1	6.3±0.2	4.7±0.6
I114A(monomer)	1.9±0.1	4.2±0.6	2.3±0.4
P116A(monomer)	1.4±0.1	10.0±0.6	7.3±0.4

a The kinetics and affinity constants were derived from 2 independent experiments similar to those shown in Figure 8. *K* values are means ± standard deviations. All the mutants were derived from the monomeric form of IL18BP with the HVEC mutation.

b NB: no binding.

Sequence analysis shows that the residues key to YLDV-IL18BP homo-dimerization (H30, P32, V33, C132) are conserved only in yatapoxvirus IL18BPs but not in other IL18BPs (Figure 1). Therefore, dimer formation and bivalent binding of IL18 seems to be unique to yatapoxvirus IL18BPs. In fact, ECTV-IL18BP and human IL18BP were reported to be monomers in solution [14], [22].

### YLDV-IL18BP:IL18 Complex Interface

**Figure 6:**
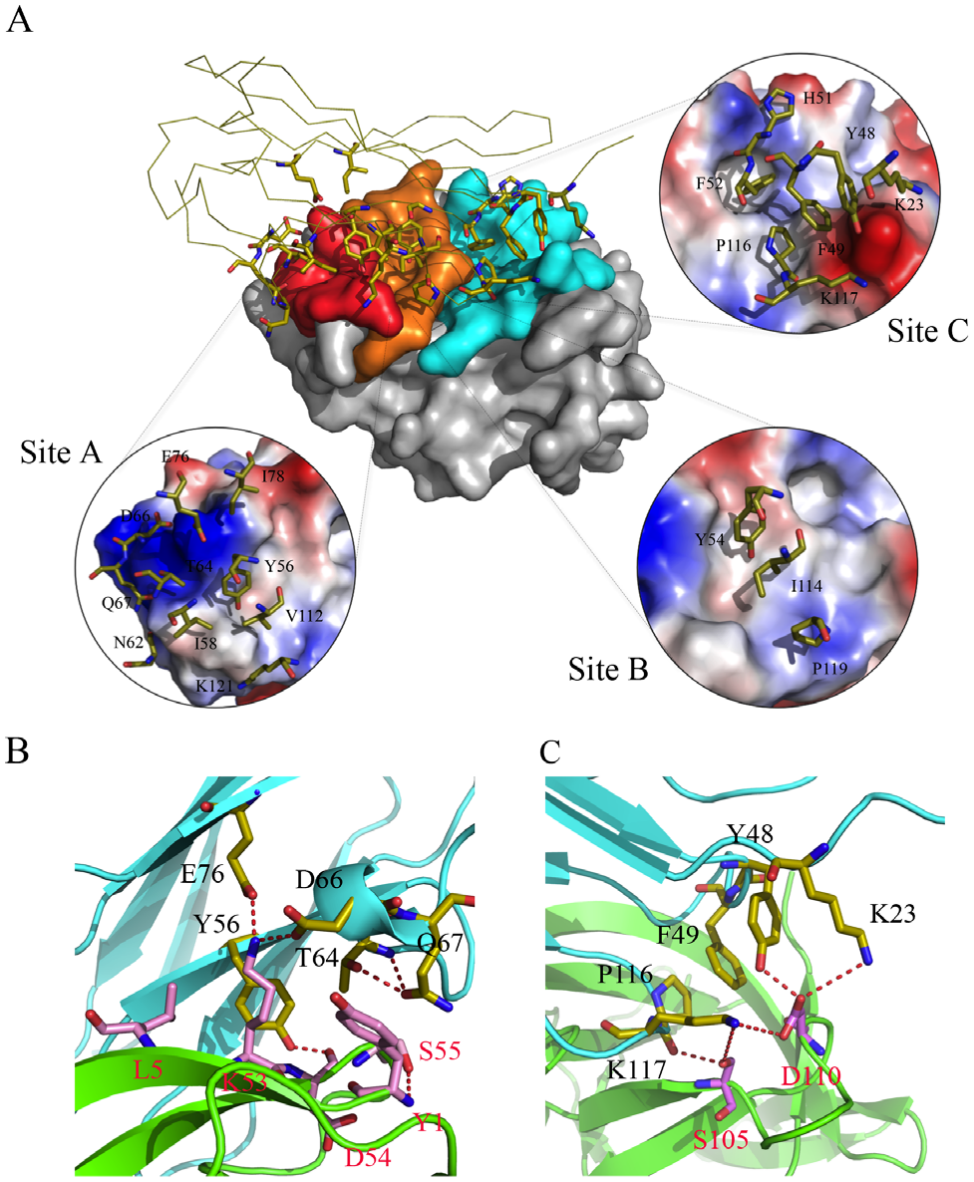
YLDV-IL18BP:IL18 interface. A). Key residues of YLDV-IL18BP at the interface. YLDV-IL18BP binds nearly identical surface of IL18 as previously observed in ECTV-IL18BP inhibitory complex. IL18 is shown as surface representation and colored grey. YLDV-IL18BP is drawn as a ribbon diagram with β-sheets colored in yellow. Binding sites A, B and C on IL18 surface are colored red, orange and cyan respectively. YLDV-IL18BP residues involved in binding IL18 are shown as stick representations. Each insert details the interactions involved in the respective binding site between YLDV-IL18BP and IL18. B). Unique interactions at binding site A. Carbon atoms of YLDV-IL18BP and IL18 are colored in yellow and pink, respectively. The secondary structures of YLDV-IL18BP and IL18 are colored in cyan and green, respectively. Red dashed lines indicate H-bonds. C). Unique interactions at binding site C. YLDV-IL18BP P116 is involved in favorable hydrophobic interactions with IL18. Polar interactions at site C involve S105 and D110 residues of IL18. The coloring scheme is the same as in B.

**Figure 7:**
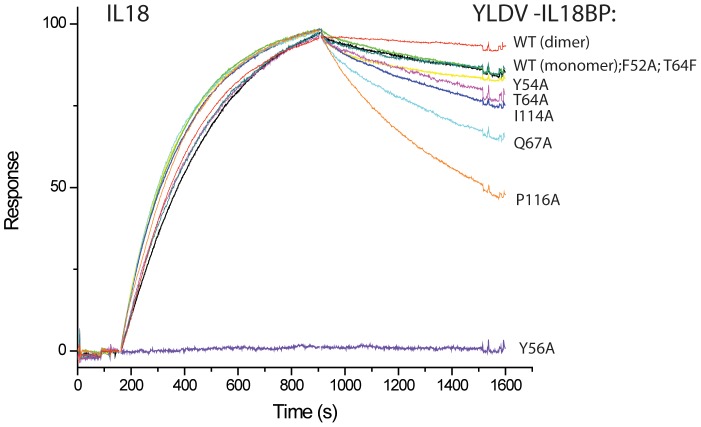
Biacore SPR Analysis of YLDV-IL18BP mutants. Biotinylated IL18 was captured on a BIAcore streptavidin-coated CM5 chip, and its binding with IL18BP was monitored with a BIAcore 3000 sensor. All the YLDV-IL18BP proteins were expressed in *E. coli* as SUMO fusion and purified to near homogeneity. The IL18BP mutants were derived from the monomeric form of the protein with the HVEC mutation. The injection of IL18BP started at ∼150 s and stopped at 900 s. The colored lines are the responses obtained with different IL18BP mutants and normalized to a maximum of 100 resonance units (RU) (except for Y56A) for ease of comparison.

**Figure 8:**
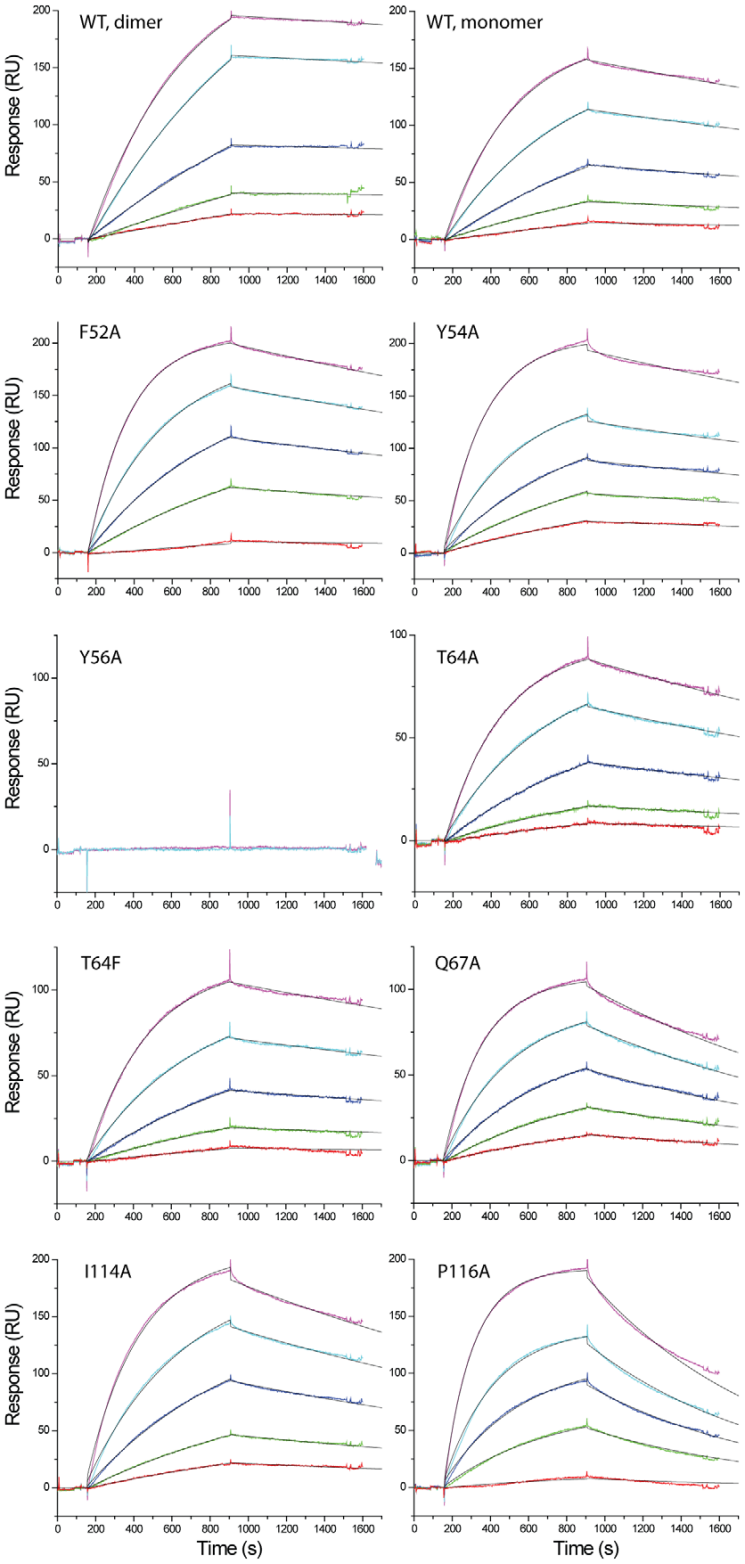
Kinetic analyses of the binding of IL18 with YLDV-IL18BP. SPR analysis was performed as described in Figure 7 with YLDV-IL18BP at 5 different concentrations. The binding curves were globally fitted with BiaEvaluation software to a 1∶1 binding model. The colored and black lines are the actual responses in RU and globally fitted curves, respectively.

**Figure 9:**
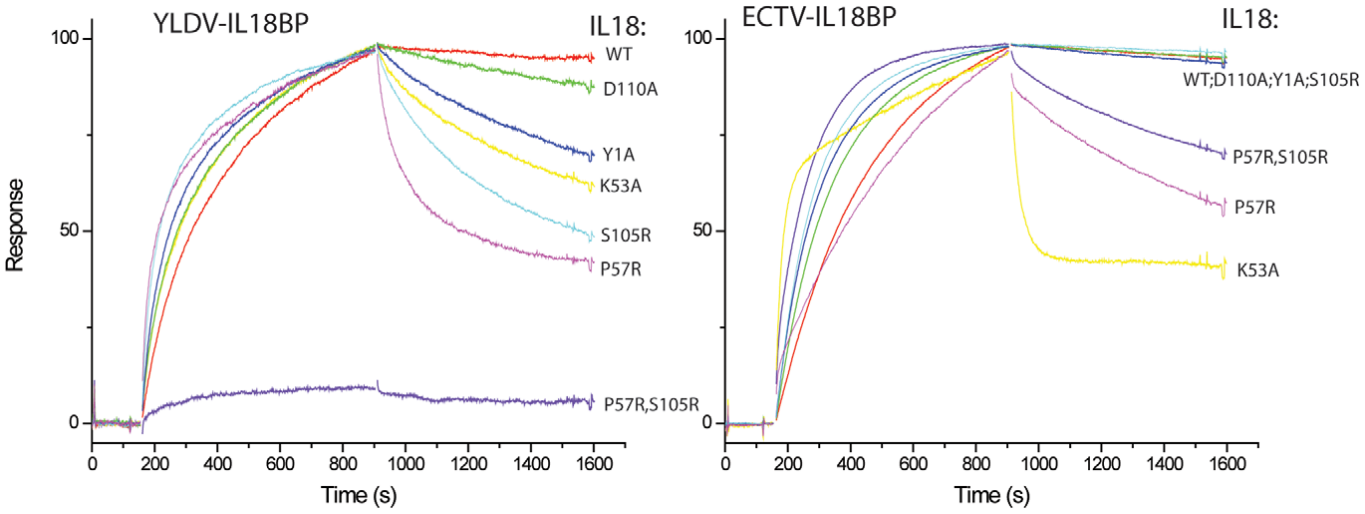
Biacore SPR analysis of IL18 mutants. Biotinylated YLDV-IL18BP and ECTV-IL18BP were captured on two different flow cells in a BIAcore streptavidin-coated CM5 chip, and their binding with IL18 was monitored simultaneously with a BIAcore 3000 sensor. The injection of IL18 started at ∼150 s and stopped at 900 s. The colored lines are the responses obtained with different IL18 mutants and normalized to a maximum of 100 RU (except for P57R, S105R for YLDV-IL18BP) for ease of comparison. YLDV-IL18BP and ECTV-IL18BP were expressed and secreted from mammalian cells and underwent *in vitro* biotinylation as described in Materials and Methods.

**Figure 10:**
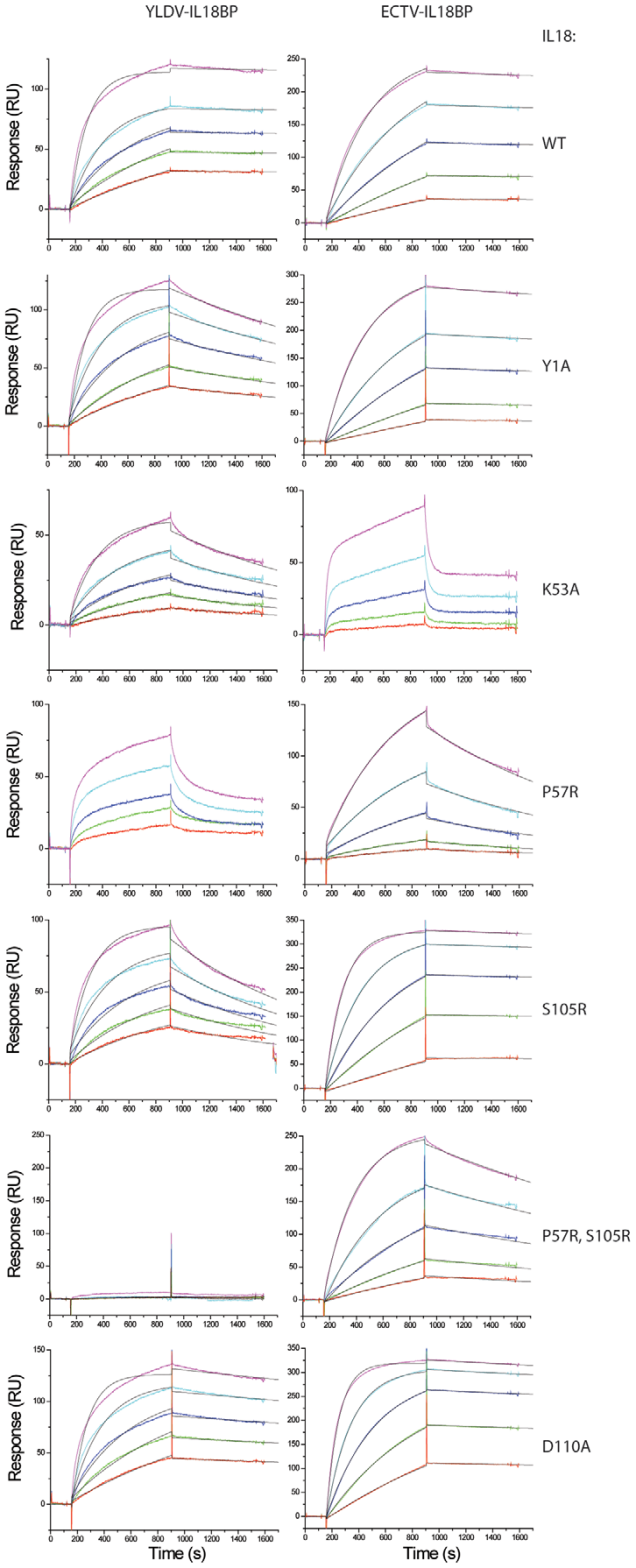
Kinetic analyses of the binding of IL18 with YLDV-IL18BP or ECTV-IL18BP. SPR analysis was performed as described in Figure 9 with IL18 at 5 different concentrations. The binding curves were globally fitted with BiaEvaluation software to a 1∶1 binding model. The colored and black lines are the actual responses in RU and globally fitted curves, respectively.

YLDV-IL18BP binds IL18 by using the same edge of the β sandwich as observed in ECTV-IL18BP:IL18 complex structure. Specifically, the following regions on YLDV-IL18BP are observed at the interface: loop connecting βB–βC, βC, the short βD, helix H1, βG, and loop connecting βG–βH (Figures 2,3). Similar to what was observed in the ECTV-IL18BP:IL18 complex structure, YLDV-IL18BP molecule sits atop the opening of the IL18 β-barrel and binds the cytokine through extensive hydrophobic and hydrogen bonding interactions (Figure 6), covering about 1,957 Å^2^ of ASA, which is comparable to the ECTV-IL18BP:IL18 complex at 1,930 Å^2^ ASA as identified by the program AreaIMol of the CCP4 suite [23]. The numbers of residues involved at binding interfaces are also comparable between the two inhibitory complexes. YLDV-IL18BP contributes mainly 19 residues to the complex interface while IL18 contributes 23 residues, in comparison to 17 residues from ECTV-IL18BP and 25 residues from IL18 using NCont of the CCP4 suite [23]. To assess the energetic contributions to binding by residues at the binding interface, we performed site-directed mutagenesis on both IL18 and the monomeric YLDV-IL18BP (HVEC) and assessed the effects of the mutations on the binding affinity by SPR (Figures 7,8,9,10). In addition, to probe the difference in IL18 binding by IL18BPs of YLDV and ECTV, we performed binding studies of various IL18 mutants with the two IL18BPs simultaneously (Figures 9,10). We will describe the results from these functional studies in context of our depiction of the YLDV-IL18BP:IL18 complex interface. As we described in the previous ECTV-IL18BP:IL18 complex structure, we will continue to use the three identified binding sites, labeled as A, B and C on IL18 here (Figure 6).

Site A of IL18 contains key residues Y1, L5, K53, D54, S55 and P57, making extensive interactions with YLDV-IL18BP (Figure 6A,B). The detailed interactions are similar to those observed in the previous ECTV-IL18BP:IL18 complex structure except for the loss of one of the ‘hot spot’ interactions involving a phenylalanine (F67 in ECTV-IL18BP, described below). Substitution of S55 of IL18 with alanine was previously shown to decrease its binding affinity to orthopoxvirus IL18BP by 7-fold [18]. As observed in the ECTV-IL18BP:IL18 complex, the side chain hydroxyl of S55 is tethered to the main chain amino group of Y1 via a hydrogen bond in the YLDV-IL18BP:IL18 complex. Y56 on βC of YLDV-IL18BP occupies nearly identical position as observed in the ECTV-IL18BP complex without any conformational changes. Its phenolic group is tethered to the D54 main chain of IL18, while its aromatic side chain together with the methyl group of T64 from YLDV-IL18BP, and L5 of IL18 constructs a hydrophobic wall, entrenching the aliphatic side chain of K53 from IL18 (Figure 6B). We found mutation of Y56A on YLDV-IL18BP completely abolished its binding to IL18 (Figures 7,8, Table 2), which is consistent with previous functional analysis of other IL18BPs [16].Therefore, a tyrosine residue at this location in IL18BPs seems to be a conserved ‘hot spot’, as the most important point to anchor the inhibitory protein to IL18.

As predicted, T64 of YLDV-IL18BP, located on the tip of βD, indeed occupies the same location of F67 from ECTV-IL18BP. This phenylalanine residue is highly conserved in IL18BPs of various species including human, all orthopoxviruses and MCV. Mutations at this location of various IL18BPs were shown to dramatically reduce the binding with IL18 [15]–[17]. In the ECTV-IL18BP:IL18 complex, F67 is inserted into an induced hydrophobic pocket, forming strong interactions with IL18 residues located on the surface while forming a strong π-cation interaction with the charged head group from the side chain of K53 of IL18 [14]. These interactions are absent in YLDV-IL18BP complex due to the presence of a threonine instead of phenylalanine. Interestingly, T64F substitution in YLDV-IL18BP did not increase the binding of IL18 (Table 2, Figures 7,8), while T64A substitution only caused a 2.5-fold decrease (student t-test P-value<0.05) in binding affinity. Consistent with the lack of π-cation interaction, IL18 K53 contributes less to the binding with YLDV-IL18BP than with the orthopoxvirus IL18BPs. While K53A mutation drastically reduced the binding affinity to orthopoxvirus IL18BPs [i.e., more than 100-fold decrease for variola IL18BP [18], Figures 9 and 10], this mutation had less impact on binding affinity with YLDV-IL18BP (about 30-fold decrease, Table 3, Figures 9, 10). K53 of IL18 nevertheless remains important for binding to YLDV-IL18BP, because polar interactions involving K53 are preserved in the current structure. Specifically, the positively charged amino head group on the side chain of K53 forms salt bridges with D66 and E76 of YLDV-IL18BP, similar to the interactions of K53 with two glutamate residues of ECTV-IL18BP in the ECTV-IL18BP:IL18 complex structure.

**Table 3:** Kinetics and affinity constants of the binding of IL18 mutants with immobilized IL18BPs.

	YLDV-IL18BP			ECTV-IL18BP		
IL18 mutants^a^	K_On_,10^5^/Ms	K_off_,10^−4^/s	K_D_, nM	K_On_,10^5^/Ms	K_off_,10^−4^/s	K_D_, nM
WT	1.2±0.1	0.2±0.1	0.2±0.1	1.7±0.3	0.4±0.1	0.2±0.1
Y1A	1.1±0.2	4.5±0.5	4±1	1.7±0.5	0.5±0.1	0.3±0.1
K53A	1.1±0.1	6.3±0.8	6±1	NF^b^		
P57R	NF^b^			0.4±0.2	8±2	25±7
S105R	1.3±0.1	8.3±0.1	6.7±0.6	3.5±0.4	0.2±0.1	0.1±0.1
P57R,S105R	NB^c^			0.9±0.3	3.9±0.4	4±1
D110A	1.0±0.1	1.1±0.1	1.1±0.1	2.6±0.2	0.3±0.1	0.2±0.1

a The kinetics and affinity constants were derived from 2 independent experiments similar to those shown in Figure 10. *K* values are means ± standard deviations.

b NF: binding data did not fit 1∶1 binding model.

c NB: no binding.

Site A differences also include the side chain rotation and repositioning of Q67 on YLDV-IL18BP (equivalent to H70 of ECTV-IL18BP) and Y1 on IL18, creating a novel interaction that was absent in the ETCV-IL18BP:IL18 structure. Q67 is located in close vicinity to T64 and rotates about 90 degree (*vs.* H70 of ECTV-IL18BP) forming bifurcated hydrogen bonds with the hydroxyl group and the main chain amide nitrogen of T64. Y1 of IL18 rotates about 80 degrees and stacks on the aliphatic portion of the Q67 side chain, forming favorable van der Waals interactions (Figure 6B). Y1A substitution of IL18 and Q67A substitution of YLDV-IL18BP decreased the binding affinity by 20- and 4-fold, respectively (student t-test P-value<0.05, Table 2, 3, Figures 7,8,9,10). In contrast, Y1A mutation did not affect the affinity with ECTV-IL18BP (Table 3, Figures 9,10). Since T64A substitution of YLDV-IL18BP showed very little effect on binding to IL18 (Table 2, Figures 7,8), the hydrogen bond between the side chain of Q67 and the main chain of T64 seems to be more significant than its interaction with the side chain. It is likely Q67 further stabilizes the local structure, including helix H1 where D66 locates, allowing correct positioning of this acidic residue for interacting with K53 of IL18.

Site B is a large, predominantly hydrophobic cavity spatially adjacent to Site A on the surface of IL18 (Figure 6A). As observed in the ECTV-IL18BP complex, three non-contiguous residues, Y54, I114 and P119 (Y51, T113 and V118 in ECTV-IL18BP) from YLDV-IL18BP βC, βG and G-H loop reside but do not fully occupy the pocket. Y54A substitution in YLDV-IL18BP had negligible effects on IL18 binding, while I114A mutation caused only a 1.8-fold decrease in binding affinity (statistically not significant, student t-test P-value>0.05) (Figures 7,8, Table 2), similar to substitutions of the equivalent positions of other IL18BPs [16], [17]. Therefore, site B interactions appear not essential for binding of YLDV-IL18BP, similar to observations for other IL18BPs [14].

Site C of IL18 is next to Site B and mainly comprised of 10 IL18 surface residues involving a mixture of charged and hydrophobic interactions. Similar to what was observed in the ECTV-IL18BP complex, the loops connecting βB–βC and βG–βH of YLDV-IL18BP interact with Site C on IL18 predominantly through hydrophobic interactions. YLDV-IL18BP F52 adopts nearly identical conformation as ECTV-IL18BP F49, which is inserted into the large hydrophobic pocket of Site C [14]. Surprisingly, F52A mutation of YLDV-IL18BP had negligible effect on binding with IL18 (Figures 7,8), in contrast to the mutation at this location in other IL18BPs significantly decreasing binding affinity to IL18 (83–fold decrease for human IL-18BP:human IL18, 8-fold decrease for ECTV-IL18BP:human IL18 and 138-fold decrease for ECTV-IL18BP:murine IL18) [16], [17]. This difference can be explained by several unique interactions of YLDV-IL18BP at Site C. Residue P116 of YLDV-IL18BP is situated in a hydrophobic groove formed by aliphatic side chains from M60, Q103 and M113 of IL18 and is stabilized by a stair-wise hydrophobic stacking by side chains from F49, Y48 and K23 of YLDV-IL18BP (Figure 6A,C). In addition, P116 main chain is hydrogen bonded with the hydroxyl group from the side chain of IL18 S105, further stabilizing the complex interface. Indeed, P116A substitution reduced binding of YLDV-IL18BP to IL18 by approximately 6-fold (student t-test P-value<0.005, Figures 7,8, Table 2). Mutation of the equivalent residue in human IL18BP (P153) showed negligible effect on its binding affinity to IL18 [15], so P116 appears to be specific for YLDV-IL18BP in binding to IL18. Y48 and K23 of YLDV-IL18BP are hydrogen bonded with IL18 D110 through side chain to side chain interactions. K117 of YLDV-IL18BP forms a bifurcated hydrogen bond with side chains from IL18 S105 and D110 (Figure 6C). D110A of IL18 decreased affinity with YLDV-IL18BP by 6-fold (student t-test P-value<0.05) but had no effect on binding with ECTV-IL18BP (Figures 9,10). Similarly, S105R of IL18 caused more than 30-fold decrease (student t-test P-value<0.005) in binding affinity with YLDV-IL18BP but had no impact on binding with ECTV-IL18BP (Figures 9,10). Therefore, D110 and S105 of IL18 are specifically required for the binding of IL18 with YLDV-IL18BP but not for ECTV-IL18BP. The specificity of S105 towards binding of YLDV-IL18BP is further signified by a double mutant, S105R/P57R of IL18. This double mutant completely abolished the binding with YLDV-IL18BP, while it had a much smaller impact on binding with ECTV-IL18BP (Table 3, Figures 9,10).

## Discussion

We previously determined the crystal structure of the ectromelia virus IL18BP in complex with IL18, revealing the structural basis for the binding and inhibition of IL18 by the IL18BPs [14]. Despite an overall low sequence homology between the diverse viral and host IL18BPs, the key residues of ECTV IL18BPs at the IL18 binding interface are highly conserved. Mutagenesis studies on human and viral IL18BPs also showed that these key residues are almost universally critical for the binding of IL18BPs to IL18. Thus it was enigmatic that functional yatapoxvirus IL18BPs lack a key phenylalanine residue (Figure 1) that has been identified to be essential for many other IL18BPs to bind IL18 [14]–[17]. It is similarly puzzling that a residue of IL18 (K53) that is critical for binding orthopoxvirus IL18BPs only played a modest role in binding with the YMTV-IL18BP [13]. In this report, we resolved these questions by determining the crystal structure of YLDV-IL18BP:IL18 complex and by performing extensive mutagenesis and SPR studies. We revealed two unique signature features of YLDV-IL18BP that distinguish yatapoxvirus IL18BPs from the rest of IL18BP family members. First, YLDV-IL18BP forms a homo-dimer and interacts with IL18 in a 2∶2 binding mode. Second, the binding of YLDV-IL18BP and IL18 does not rely on two of the ‘hot spot’ interactions that were shown to be essential for the binding of all previously studied IL18BPs, including a phenylalanine (F67 in ECTV-IL18BP) at site A and another phenylalanine at site C (F49 in ECTV-IL18BP). Instead, yatapoxvirus IL18BPs evolved interactions with some IL18 residues (Y1, D110, S105) that are specifically important for binding with YLDV-IL18BP. It appears that YLDV-IL18BP shifts and disperses the binding energy across the IL18-binding interface rather than concentrating the binding energy on a few hot spots as is the case for all other IL18BPs examined to date.

It was previously reported that ECTV-IL18BP and human IL18BP are monomeric in solution [14], [22]. In contrast, YLDV-IL18BP forms a disulfide-bonded dimer, which was demonstrated not only in the crystal structure but also in solution by non-reducing SDS-PAGE and gel filtration analysis. The dimer interface is quite large (about 1,700 Å^2^) and involves extensive hydrophobic interactions in addition to the intermolecular disulfide bond, indicating that the YLDV-IL18BP dimer is intrinsically stable in solution. The dimer could only be separated into monomers by mutations that disrupt both the hydrophobic interactions as well as the inter-chain SS bond. Analysis of the monomeric YLDV-IL18BP (HVEC) showed that the dimerization was not essential for binding IL18 but enhanced the binding affinity by 3-fold in our *in vitro* assay. Although this enhancement in binding affinity as measured by SPR is modest, it is possible that the dimerization may be more important for the function of YLDV-IL18BP during infection of the host, perhaps by increasing the half-life of the protein in the infected tissue or by increasing the avidity of binding to IL18 at low protein concentration. In fact, divalent or multivalent binding is an important, inherent feature of many biological systems to enhance the effectiveness of binding of ligands to receptors and of antibodies to antigens [24]–[28]. More specifically, this has been a feature for quite a few poxvirus cytokine binding proteins. For example, ectromelia virus IFN-γ binding protein forms a tetramer, which is required for efficient IFN-γ antagonism [29]. Myxoma virus T2 protein, a Tumor Necrosis Factor (TNF) Receptor homolog, is secreted as both monomer and dimer, and the dimeric T2 is a more potent TNF inhibitor [30]. Because residues of YLDV-IL18BP involved in dimer formation are only conserved in yatapoxviruses, yatapoxviruses IL18BPs may be unique among IL18BPs in that they use bivalent binding to increase the affinity and avidity for IL18.

Another difference between YLDV-IL18BP and all other IL18BPs is the lack of two of the ‘hot spot’ interactions at the binding sites A and C on the surface of IL18. The structure of ECTV-IL18BP:IL18 complex showed that a conserved phenylalanine (F67) is engaged in hydrophobic and strong π-cation interactions with K53 of IL18 at binding site A [14]. Alanine substitutions of K53 of IL18 significant decreased binding with orthopoxvirus IL18BPs [18], while alanine substitutions of the conserved phenylalanine (equivalent to ECTV-IL18BP F67) in human, MCV and orthopoxviruses IL18BPs significantly decreased or completely abolished binding of IL18 [15]–[17]. The current structure of YLDV-IL18BP:IL18 complex showed that a threonine residue (T64) is present at the position equivalent to the phenylalanine, indicating the π-cation interaction with K53 is not important for YLDV-IL18BP: IL18 complex. Indeed, T64F or T64A substitution of YLDV-IL18BP had negligible or minor (2.5-fold decrease in affinity for T64A, student t-test P-value<0.05) effect on the binding with IL18, while K53A of IL18 had a more modest effect on binding with YLDV-IL18BP than with orthopoxvirus IL18BPs. A similar loss of ‘hot spot’ interaction was also observed at binding site C. A phenylalanine residue on IL18BPs that binds to site C of IL18 was previously shown to be important for binding IL18 in orthopoxvirus, MCV and human IL18BPs [15]–[17]. Although a phenylalanine (F52) is present at the equivalent position in YLDV-IL18BP, it is not important for binding to IL18 (Table 2, Figures 7,8). Through structural and mutagenesis studies, we have identified contact residues that are unique to the YLDV-IL18BP:IL18 binding interface. This includes Q67 of YLDV-IL18BP and Y1 of IL18 at site A, P116 of YLDV-IL18BP and S105 and D110 of IL18 at site C. Our data are in agreement with the conclusion of a more delocalized energy distribution for binding of IL18 to YMTV-IL18BP [13]. The structural and functional studies of two different IL18BP complexes suggest that there is a degree of plasticity in the IL18BP:IL18 interface that could accommodate certain mutations in IL18BPs without compromising their binding affinity to IL18.

Despite the differences in several key residues for binding IL18, the current YLDV-IL18BP:IL18 complex structure showed that YLDV-IL18BP targets the same surface of IL18 as ECTV-IL18BP does in the previous complex structure. This suggests that all IL18BPs inhibit IL18 function by blocking a putative receptor-binding site on the surface of IL18. Similar to previous findings on human and poxvirus IL18BPs, Y56 of YLDV-IL18BP (interacting with site A of IL18) was found to be absolutely essential for binding to IL18, indicating that this conserved tyrosine residue is an obligatory ‘anchor’ for binding of all IL18BPs to IL18. The conservation and variation in functional residues and their specific interactions with IL18 suggest that IL18BPs share a common ancestor but may have undergone significant evolution through different selection pressures, resulting in a conserved inhibitory mechanism albeit with mutations of interface residues.

The biological activity of IL18 is determined in part by its relative affinities for IL18 receptors and IL18BP. The binding of IL18 to its receptors triggers multiple cellular responses vital to immunity, but excessive IL18 activities are associated with many autoimmune and inflammatory diseases [4], [31]–[37]. Functional IL18BPs are present in many poxviruses including variola virus and vaccinia virus, providing a key strategy of poxvirus immune evasion by inhibition of IL18 cytokine activity. Therefore the studies on IL18BP:IL18 inhibitory complexes could serve dual purposes by providing important clues on how to develop functional inhibitors targeting either IL18 or poxvirus IL18BP. These inhibitors could potentially modulate IL18 and poxvirus IL18BP activities, which may benefit efforts in developing treatments against some autoimmune and inflammatory diseases and in developing treatments for potential pathogenic outbreaks associated with poxvirus infections.

## Materials and Methods

### Protein Purification and Crystallization

Mature IL18 and YLDV-IL18BP (residues 20–136) were individually cloned into a modified pET vector as SUMO fusion proteins with N-terminal 6×His tags and expressed in *E. coli* BL21 (DE3) gold (*Stratagene*) or Rosetta-Gami 2 (*Invitrogen*) strains, respectively. An IL18 mutant (C38S, C68S, C76S, C127S, K67A, E69A, K70A, I71A) with substitutions of four nonessential cysteines [20] and four additional surface residues opposite to the IL18BP binding interface, IL18 (8S), and the triple-cysteine mutant of YLDV-IL18BP (residues 22–136, C87S, C132S) were cloned and expressed in the same way as WT proteins. The individual proteins were purified using the similar double Ni-nitrilotriacetic acid (Ni-NTA) procedure as described [14]. Briefly, the his-tagged fusion proteins were first purified from cell lysate by Ni-NTA affinity column (*Qiagen*) and then co-dialyzed with ULP1 protease to remove the SUMO moiety, exposing the authentic N-terminus for both proteins. The cleaved protein mixtures were subsequently passed through a second subtracting Ni-NTA column and further purified by size exclusion chromatography on a Superdex s200 column. The YLDV-IL18BP and IL18 (8S) proteins were mixed together and the complexes were subsequently purified from size exclusion chromatography and each concentrated to 9 mg/ml. The complex of wild-type YLDV-IL18BP:IL18 (8S) crystallized in a condition containing 18% PEG3350, 0.1 M Bis-Tris Propane/Citric Acid, pH 6.5, while the crystallization condition for the complex of triple-cysteine mutant YLDV-IL18BP:IL18 (8S) is 12% PEG3350, 0.1 M Tris, pH 8.0. 25% ethylene glycol was added step-wise to the mother liquid as cryoprotectant.

To make biotinylated IL18, mature human IL18 (residues 37–193) was cloned into a modified pET vector containing a C-terminal 6×His tag along with the coding sequence (GLNDIFEAQKIEWHE) for biotinylation. This plasmid and a plasmid encoding biotin ligase (*Avidity*) were co-transformed into *E. coli BL21* (DE3) gold (*Stratagene*) for expression. One step Ni-NTA affinity purification was used to purify biotinylated and his-tagged IL18.

### Mammalian Expression, Purification and In Vitro Biotinylation of IL18BPs

For mammalian expression of YLDV-IL18BP, a mammalian expression plasmid for YLDV 14L was constructed as described previously for the construction of expression vector for human IL18BP [16]. Briefly, YLDV 14L was amplified by PCR from genomic DNA of YLDV and cloned into pYX45 with NheI and BamHI sites, so that 14L ORF was appended with a C-terminal biotinylation tag and 6-His tag. 293T cells were transfected with the expression plasmid and then infected with vTF7.3, a vaccinia virus expressing T7 polymerase. 3 days later, the medium was harvested and incubated with Ni-NTA resin (*Qiagen*). The resin was then washed and added with *E. coli* biotin holoenzyme synthetase (*Avidity*). After the biotinylation reaction, the protein was eluted with phosphate-buffered saline containing 300 mM imidazole. The ECTV-IL18BP was expressed in HEK293T cells, purified from the culture medium and biotinylated essentially as described previously [18].

### Structure Determination

An initial set of data for the tripe-cysteine mutant YLDV-IL18BP:IL18 (8S) was collected at beamline X29 of National Synchrotron Light Source, Brookhaven National Laboratory. Initial phases were determined by molecular replacement using Phaser [38] of the CCP4 suite [23] and a search model containing IL18 along with a trimmed poly-alanine model of the ECTV-IL18BP (PDB ID 3F62). A subsequent data set from a crystal of WT YLDV-IL18BP:IL18 (8S) complex was collect at beamline 19-ID of the Advanced Photon Source, Argonne National Laboratory. The structure of the complex containing WT YLDV-IL18BP was solved similarly as the structure of the complex containing the triple-cysteine mutant YLDV-IL18BP. All datasets were processed with HKL3000 [39]. PHENIX [40] was used for refinement and Coot [41] was used for iterative manual model building. Translation, libration and screw-rotation displacement (TLS) groups used in the refinement were defined by the TLMSD server [42]. The structure of the triple-cysteine mutant YLDV-IL18BP:IL18 (8S) complex was refined to 1.75 Å resolution with R_work_ and R_free_ of 19.0% and 23.1% respectively. The structure of the WT YLDV-IL18BP:IL18 (8S) complex was refined to 2.7 Å resolution with R_work_ and R_free_ of 21.9% and 27.0% respectively. The final models are of good refinement statistics for both complexes as shown in Table 1. All molecular graphic figures were generated with PYMOL [43].

### Surface Plasmon Resonance

The SPR analysis was done essential as described previously [15], [16]. Briefly, biotinylated IL18BP [∼300 resonance units (RU)] or IL18 (∼250 RU) was captured onto a BIAcore CM5 chip coated with streptavidin. Various concentrations of IL18 (from ∼1 nM to ∼40 nM) or IL18BP (from ∼2 nM to ∼60 nM) were injected at a flow rate of 20 µl/min. The chip coated with IL18BP was regenerated with a 10-µl injection of 1 M NaCl, 50 mM NaOH, while the chip coated with IL18 was regenerated with a 10-µl injection of 10 mM glycine (pH 2.5). The sensorgrams were analyzed with BIAEVALUATION software (BIACORE). The binding data from the injection of at least five different concentrations of analyte were globally fitted to a 1∶1 binding model. Analyses with the same concentration series were done twice.

### Accession Codes

Protein Data Bank: structure factors and atomic coordinates for the WT YLDV-IL18BP:IL18 (8S) and the triple-cysteine mutant YLDV-IL18BP:IL18 (8S) complexes have been deposited with accession codes 4EEE and 4EKX, respectively.
